# The effects of intranasal oxytocin on reward circuitry responses in children with autism spectrum disorder

**DOI:** 10.1186/s11689-018-9228-y

**Published:** 2018-03-27

**Authors:** R. K. Greene, M. Spanos, C. Alderman, E. Walsh, J. Bizzell, M. G. Mosner, J. L. Kinard, G. D. Stuber, T. Chandrasekhar, L. C. Politte, L. Sikich, G. S. Dichter

**Affiliations:** 1 Department of Psychology and Neuroscience, University of North Carolina at Chapel Hill, Chapel Hill, NC 27514 USA; 2 Duke Clinical Research Institute, Duke University, Durham, NC 27705 USA; 3 Duke Center for Autism and Brain Development, Duke University, Durham, NC 27705 USA; 4 Department of Psychiatry, University of North Carolina at Chapel Hill School of Medicine, Chapel Hill, NC 27514 USA; 5 Duke-UNC Brain Imaging and Analysis Center, Duke University Medical Center, Durham, NC 27705 USA; 6 Carolina Institute for Developmental Disabilities, University of North Carolina at Chapel Hill School of Medicine, Chapel Hill, NC 27514 USA; 7 Department of Psychiatry and Behavioral Sciences, Duke University Medical Center, Durham, NC 27705 USA; 8 Department of Cell Biology and Physiology, University of North Carolina at Chapel Hill School of Medicine, Chapel Hill, NC 27514 USA; 9 Neuroscience Center, University of North Carolina at Chapel Hill School of Medicine, Chapel Hill, NC 27514 USA; 10 Department of Psychiatry, University of North Carolina at Chapel Hill School of Medicine, CB 7155, Chapel Hill, NC 27599-7155 USA

**Keywords:** Autism spectrum disorder, Oxytocin, Reward, fMRI

## Abstract

**Background:**

Intranasal oxytocin (OT) has been shown to improve social communication functioning of individuals with autism spectrum disorder (ASD) and, thus, has received considerable interest as a potential ASD therapeutic agent. Although preclinical research indicates that OT modulates the functional output of the mesocorticolimbic dopamine system that processes rewards, no clinical brain imaging study to date has examined the effects of OT on this system using a reward processing paradigm. To address this, we used an incentive delay task to examine the effects of a single dose of intranasal OT, versus placebo (PLC), on neural responses to social and nonsocial rewards in children with ASD.

**Methods:**

In this placebo-controlled double-blind study, 28 children and adolescents with ASD (age: *M* = 13.43 years, SD = 2.36) completed two fMRI scans, one after intranasal OT administration and one after PLC administration. During both scanning sessions, participants completed social and nonsocial incentive delay tasks. Task-based neural activation and connectivity were examined to assess the impact of OT relative to PLC on mesocorticolimbic brain responses to social and nonsocial reward anticipation and outcomes.

**Results:**

Central analyses compared the OT and PLC conditions. During nonsocial reward anticipation, there was greater activation in the right nucleus accumbens (NAcc), left anterior cingulate cortex (ACC), bilateral orbital frontal cortex (OFC), left superior frontal cortex, and right frontal pole (FP) during the OT condition relative to PLC. Alternatively, during social reward anticipation and outcomes, there were no significant increases in brain activation during the OT condition relative to PLC. A Treatment Group × Reward Condition interaction revealed relatively greater activation in the right NAcc, right caudate nucleus, left ACC, and right OFC during nonsocial relative to social reward anticipation during the OT condition relative to PLC. Additionally, these analyses revealed greater activation during nonsocial reward outcomes during the OT condition relative to PLC in the right OFC and left FP. Finally, functional connectivity analyses generally revealed changes in frontostriatal connections during the OT condition relative to PLC in response to nonsocial, but not social, rewards.

**Conclusions:**

The effects of intranasal OT administration on mesocorticolimbic brain systems that process rewards in ASD were observable primarily during the processing of nonsocial incentive salience stimuli. These findings have implications for understanding the effects of OT on neural systems that process rewards, as well as for experimental trials of novel ASD treatments developed to ameliorate social communication impairments in ASD.

**Electronic supplementary material:**

The online version of this article (10.1186/s11689-018-9228-y) contains supplementary material, which is available to authorized users.

## Background

Autism spectrum disorder (ASD) is a neurodevelopmental disorder characterized by impairments in social communication and interaction, as well as restricted and repetitive behaviors (APA [1]). Although various pharmacological treatments are commonly prescribed to treat associated symptoms of ASD (e.g., irritability, inattention, and aggression), there are currently no pharmacological treatments approved to treat the core features of the disorder [2–4].

The neuropeptide oxytocin (OT) has been shown to increase pro-social behaviors in human studies and in preclinical model organisms. Studies in typically developing individuals have shown that intranasal OT administration increases in-group trust [5] and interoceptive awareness [6] while also reducing fear [7]. Preclinical studies, on the other hand, have established the vital role of OT in sociality. For example, in mammalian nonhuman models, OT moderates or initiates paternal and reproductive behaviors, as well as other pro-social behaviors such as grooming and social recognition [8, 9].

Because of the need for effective treatments for core ASD symptoms, there has been increasing interest in the potential for OT to ameliorate social communication impairments in ASD. Some, but not all, studies of the effects of OT in ASD have reported benefits in social functioning, including enhanced emotion recognition [10], increased eye gaze [11], and enhanced feelings of trust in others [12]. Other studies, however, have failed to find clinical benefits of OT on primary social outcome measures [13, 14], and a recent trial found that the beneficial effects of OT on social functioning in ASD were moderated by pre-treatment endogenous OT levels, suggesting that OT may be beneficial for some, but not all, individuals with ASD [15].

Although there is emerging evidence that OT may be clinically beneficial for at least a significant subset of individuals with ASD, the mechanisms of action of OT are not well understood. One potential mechanism of action may be the capacity of OT to modulate sensitivity to, and the perceived salience of, external rewards that influence behavior and facilitate reward-based learning. Preclinical studies implicate the mesocorticolimbic dopamine system as a mechanism by which OT exerts its pro-social effects [16, 17]. This neural network is comprised of midbrain structures (the ventral tegmental area (VTA) and substantia nigra), the striatum, and cortical regions including the orbital frontal, anterior cingulate, and prefrontal cortices [18]. OT and mesocorticolimbic dopamine interact in such a manner that the activation of OT-responsive neurons in the VTA increases dopaminergic activity in the broader mesocorticolimbic system [19–21]. Furthermore, when administered an OT receptor agonist, mice demonstrate a subsequent decrease in dopaminergic release within the nucleus accumbens, reflecting the influence of OT on mesocorticolimbic dopamine transmission [19].

To date, no functional neuroimaging study has examined the effects of OT on the mesocorticolimbic system in response to rewards in ASD. However, two functional neuroimaging studies indicate the relevance of mesocorticolimbic brain regions to the potential mechanisms of action of OT in ASD. Gordon et al. [22] found increased activation in the ventral striatum, left posterior superior temporal sulcus, and left premotor cortex in ASD in response to acute intranasal OT administration during a socio-emotional recognition task and that these same brain regions showed decreased activation to nonsocial (i.e., object) judgements. Other research from this group found that intranasal OT administration increased functional connectivity between the ventral striatum and ventromedial prefrontal cortex in ASD in response to a biological motion task, underscoring the potential centrality of mesocorticolimbic brain regions to the mechanism of action of OT [23].

Although both of these studies highlight the potential relevance of reward-responsive mesocorticolimbic brain regions to the mechanism of action of OT in ASD, neither used a reward task to directly test this hypothesis. Thus, the goal of the present study was to extend these findings by assessing the impact of acute intranasal OT administration on response to rewards in ASD using social and nonsocial incentive delay tasks. Social and nonsocial incentive delay tasks have been used in multiple studies to investigate reward processing in ASD (for a review see [24]). These studies have consistently revealed reduced ventral striatal activation during social and nonsocial reward anticipation in ASD [25–28]. Although the pattern of mesocorticolimbic responses to rewards in ASD is complex (i.e., different studies with different sample characteristics have reported decreased ventral striatal responses to social, but not nonsocial, reward anticipation in ASD [27, 29] whereas others have reported decreased ventral striatal responses to nonsocial, but not social, reward anticipation in ASD [26]), it is clear that mesocorticolimbic responses to rewards in ASD are impaired and that incentive tasks are suitable to study the functional integrity of this system.

Participants in the current study completed functional neuroimaging scans after double-blind administration of OT or PLC, and responses to nonsocial and social rewards were examined. We hypothesized that intranasal OT administration, relative to PLC, would result in greater activation and connectivity within mesocorticolimbic brain regions (frontal lobes, amygdala, nucleus accumbens (NAcc), insula, thalamus, caudate nucleus, anterior cingulate cortex (ACC), and putamen) that have previously been found to be functionally impaired during reward processing in ASD [30]. We also hypothesized that the effects of OT would be more pronounced in the social, relative to nonsocial, reward context because of the putative pro-social effects of OT described earlier [22, 23]. Finally, we explored relations between neural response to OT, symptom severity, and salivary OT concentrations.

## Methods

### Participants

This protocol was approved by the Institutional Review Boards at the University of North Carolina at Chapel Hill and Duke University Medical Center, and informed consent was obtained from the parent or guardian of each participant before testing. Participants older than 11 also provided verbal and written assent. Participants were recruited through the Autism Research Registry maintained through the Carolina Institute for Developmental Disabilities. Exclusion criteria included a history of medical conditions associated with ASD, including Fragile X syndrome, tuberous sclerosis, neuro-fibromatosis, phenylketonuria, epilepsy and traumatic brain injury, full-scale intelligence < 70, and MRI contraindications.

The study enrolled 33 children and adolescents with ASD ages 10 to 17 years old. Diagnoses were based on a history of clinical diagnosis confirmed by proband assessment by a research reliable assessor via Module 3 or 4 of the Autism Diagnostic Observation Schedule, Second Edition (ADOS-2; [31]) using standard clinical algorithm cutoffs. Of the 33 individuals enrolled, data from 28 were analyzable (see Table 1): one participant elected to discontinue testing during the first visit, another was unable to complete the scan due to claustrophobia, and three participants were excluded due to excessive motion (see “Motion Correction” for details).

**Table 1:** Participant characteristics

Characteristic	Mean	Standard deviation	Range
Age	13.43	2.36	10–17
Full-scale IQ	103.55	15.19	75–128
ADOS-2 calibrated severity score	8.46	1.29	7–10
SRS total *T* score	76.19	10.66	49–90
Sex	26 males, 2 females		

ADOS-2 calibrated severity scores were calculated for modules 3 and 4 using guidelines established by Gotham et al. [83] and Hus and Lord [84]

*ADOS-2* Autism Diagnostic Observation Schedule, Second Edition

After providing informed consent, participants completed two fMRI sessions (one after OT administration and one after PLC administration, with the order of scans counter-balanced across participants). The two scan sessions were scheduled at least 72 h apart to minimize the possibility of carry-over effects of OT administration (mean time between scans = 15 days; range = 3–46 days). Participants were offered the opportunity to participate in an optional mock scan prior to the neuroimaging sessions. Families were compensated $50 for each visit attended.

### Drug protocol

Oxytocin (Syntocinon®, Novartis, Switzerland) and a matched solution containing no medication (PLC) were repackaged into identically appearing bottles. The administration sequence was counter-balanced by UNC Investigational Drug Service and Triangle Compounding Pharmacy, and OT and PLC were administered to participants by a blinded research assistant. A 24 international unit (IU)/mL dose of each solution was administered in alternating nostril insufflations (six total puffs) over the course of several minutes. This dose was the same as those used in multiple previous studies examining the effect of OT in adults, adolescents, and children with ASD [10, 11, 14, 22, 23]. Recent clinical and preclinical findings have demonstrated intranasal OT’s ability to increase peripheral (i.e., cerebrospinal fluid, plasma) OT concentrations [32], while preclinical research has reported augmented brain OT levels following intranasal OT administration [33–35].

### fMRI task

As described in Richey et al., participants completed two versions of an incentive delay tasks [36] such that nonsocial rewards (i.e., money) and social rewards (i.e., pictures of smiling faces) were presented as rewards on alternating runs. Participants were presented with two runs of the nonsocial reward condition and two runs of the social reward condition. On all runs, rewards could be won or not won (i.e., there was no “loss” condition). Face stimuli were smiling images from the NimStim set of facial expressions [37]. Each run began with a 10-s instructional screen indicating the forthcoming reward type (i.e., nonsocial or social), and the two task types were segregated by run to minimize the number of cues to be memorized.

Each trial consisted of (1) a 2000-ms cue indicating whether adequately quick responses to the bull’s-eye would result in a “win” (a triangle) or not (a circle); (2) a 2000–2500-ms crosshair fixation; (3) a target bull’s-eye presented for up to 500 ms that requires a speeded button press; (4) 3000 ms of feedback that indicated whether that trial was a “win” or not, with wins accompanied by either an image of money or a face; and (5) a variable length ITI crosshair resulting in a total trial duration of 12 s. Potential win and non-win trials were aperiodic and pseudorandomly ordered. Each 8-min run contained 40 trails, half of which were potential win trials. The task was adaptive such that participants were successful on two thirds of trials, regardless of individual differences in RTs (confirmed via inspection of behavioral data collected during scanning). Mean reaction times were calculated during practice trials prior to the scan and then entered into the fMRI paradigm to ensure that participants succeeded on 66% of their responses as described in [36].

During nonsocial runs, participants won $1 per trial if bull’s-eye responses were adequately quick. During social runs, participants viewed a face image if bull’s-eye responses were adequately quick. Coincident with feedback, cumulative win totals were presented. Participants were instructed to respond to all target bull’s-eyes as quickly as possible to win on as many trials as possible and win or non-win outcomes were contingent on reaction times (RTs). Standard administration of incentive delay tasks involves showing participants’ rewards that may be won prior to scanning [36]. Consistent with this procedure, participants were shown the money they could win based on scanner task performance and were informed that they would receive the total amount of money won during the scan. Prior to scanning, participants rated face stimuli on the dimensions of valence and arousal. Stimuli were presented using E-Prime presentation software version 2.0 (Psychology Software Tools Inc., Pittsburgh, PA, USA).

Prior to and immediately following each scan, participants were asked to rate face stimuli on the dimensions of valence, arousal, and trust using Qualtrics software (Qualtrics, Provo, UT) on a computer outside of the scanner (pre-scan ratings were completed prior to the nasal spray administration).

### Imaging methods and preprocessing

Functional imaging data were acquired at the Duke-UNC Brain Imaging and Analysis Center (BIAC) on a 3.0-T General Electric (Waukesha, WI, USA) MR750 scanner system equipped with 50 mT/m gradients and an eight-channel head coil. High-resolution T1-weighted anatomical images were acquired with 256 axial slices using an FSPGR pulse sequence (TR = 8.16 ms, TE = 3.18 ms; flip angle = 12°; FOV = 256; image matrix = 256 mm^2^; voxel size = 1 × 1 × 1 mm) for normalization and co-registration. Whole brain functional images were acquired with 64 axial slices oriented parallel to the AC-PC plane using a spiral-in SENSE sequence (TR = 1500 ms, TE = 30 ms; flip angle = 60°; FOV = 240; image matrix = 64 mm^2^; voxel size = 3.75 × 3.75 × 4 mm). The first four volumes of each functional task were discarded to allow for steady state equilibrium.

Functional data were preprocessed using FSL version 5.0.1 (Oxford Centre for Functional Magnetic Resonance Imaging of the Brain (FMRIB), Oxford University, UK). Preprocessing was applied as follows: (1) brain extraction for non-brain removal [38], (2) motion correction using MCFLIRT [39], (3) spatial smoothing using a Gaussian kernel of FWHM 5 mm, (4) mean-based intensity normalization of all volumes by the same factor, and (5) high-pass filtering [40]. Functional images were co-registered to structural images in native space, and structural images were normalized into a standard stereotaxic space (Montreal Neurological Institute). Registrations used an intermodal registration tool [38, 40]. Voxel-wise temporal autocorrelation was estimated and corrected using FMRIB’s Improved Linear Model [41].

#### Motion correction

Consistent with motion thresholds used in Gordon et al. [22], runs with maximum motion > 3 mm along any of six axes (i.e., *x*, *y*, *z*, pitch, yaw, and roll) were excluded from analyses. Due to excessive motion (> 3 mm), some participants only had one social and/or nonsocial reward condition run per scan. Participants were only included in the final analyses if they had at least one nonsocial and one social run that met motion criteria for both their OT and PLC scans. Either due to motion or the participant’s ability to stay in the scanner for the entire length of the scan, 17 of the 56 scans had less than four total runs. Sixty-six percent of runs included in analyses had < 1.0 mm of motion in any axis (pitch, roll, yaw, *x*, *y*, *z*), 26% had 1.0–1.99 mm of motion, and 8% had motion between 2.0 and 2.9 mm. In addition to conducting motion correction using MCFLIRT [39], time points with large motion, as defined by FSL, were entered into the general linear model (GLM) model as additional confound variables within first-level analyses using FSL’s motion outlier detection program (http://fsl.fmrib.ox.ac.uk/fsl/fslwiki/FSLMotionOutliers). Following motion correction, paired *t* tests were used to compare differences in motion between OT and PLC groups: there was equivalent motion for mean and maximum values along all six axes (i.e., *x*, *y*, *z*, pitch, yaw, and roll), all *p* values > .05.

#### fMRI analysis

Planned analyses included (1) treatment group (OT vs. PLC) differences in frontostriatal functional activation and connectivity in response to social reward anticipation and outcomes; (2) treatment group differences in frontostriatal functional activation and connectivity in response to nonsocial reward anticipation and outcomes; (3) treatment group differences in frontostriatal functional activation in response to nonsocial relative to social reward anticipation and outcomes, conducted also with a small volume correction for the striatum alone given the centrality of this region for reward processing; and (4) correlations between frontostriatal functional activation and connectivity with ASD symptoms and salivary OT analyses.

Supplemental analyses included (1) main effects of OT and PLC separately on whole brain functional activation in response to nonsocial and social reward anticipation and outcomes, (2) treatment group (OT vs. PLC) differences in frontostriatal structural activation in response to social and nonsocial reward anticipation and outcomes, (3) correlations between structural activation with ASD symptoms, and (4) treatment group differences in frontostriatal functional connectivity of structurally defined clusters in response to social and nonsocial reward anticipation and outcomes.

#### Small volume corrections

For all analyses, anticipation and outcome phases were analyzed separately. Key anatomical regions within the reward system (superior frontal gyrus, medial frontal gyrus, orbitofrontal gyrus, paracingulate gyrus, amygdala, nucleus accumbens (NAcc), insula, thalamus, caudate nucleus, anterior cingulate cortex (ACC), and putamen) were defined a priori for small volume correction. These regions were generated separately for the right and left hemispheres in FSL using the Harvard–Oxford cortical and subcortical structural probabilistic atlases. Masks were thresholded at 25%, binarized, and then combined into a single mask using fslmaths. For planned main effect analyses (i.e., nonsocial and social reward conditions analyzed independently) and planned interaction analyses (i.e., nonsocial > social, social > nonsocial), voxels were considered significant if they passed a threshold of *p* < .005 and were part of a 39-voxel cluster of contiguous significant voxels, resulting in a cluster-corrected *p* < .05. This cluster size was determined by performing 1000 Monte Carlo simulations using 3dClustSim [42]. Interaction analyses (e.g., nonsocial > social) also included an analysis using a small volume correction that included only the striatum given our a priori interest in the striatum. Due to this small volume correction, interaction clusters within the striatum were considered significant if they passed a statistical threshold of *p* < .005 and were part of a 17-voxel cluster of contiguous significant voxels, resulting in a cluster-corrected threshold of *p* < .05 (again determined by performing 1000 Monte Carlo simulations using 3dClustSim [42]). Localizations were based on Harvard–Oxford cortical and subcortical structural probabilistic atlases as implemented in FSLView version 5.0.1, and all activations were visualized with MRIcron (https://www.nitrc.org/projects/mricron/).

#### Activation analyses

Whole brain general linear model (GLM) activation analyses were conducted using the FSL expert analysis tool (FEAT). For ROI analyses, each participant’s condition-specific mean percent signal change was calculated for both the social and nonsocial conditions. Within-participant activation differences were analyzed for treatment effects using paired *t* tests and using a 2 (Treatment Group: OT, PLC) × 2 (Reward Condition: nonsocial, social) ANOVA (see Additional file 1: Supplementary Materials). Structural ROI activation results are also provided in Additional file 1: Supplementary Materials.

#### Connectivity analyses

Task-based functional connectivity was analyzed using a generalized psychophysiological interaction (gPPI) approach due to its improved power, sensitivity, and specificity in detecting context-dependent functional connectivity [43, 44]. Functional seeds were derived from activation clusters showing significant OT > PLC effects. These seeds were supplemented with structural left and right NAcc seeds because of the centrality of the NAcc to the mesocorticolimbic reward processing system [36], once again defined using the Harvard–Oxford subcortical structural probabilistic atlas. Voxel-wise models evaluated whole-brain connectivity with these seeds. For each participant, mean fMRI time courses (i.e., physiological regressors) were extracted from seed regions for each task run using *fslmeants* in FSL, then multiplied by each psychological regressor of interest (i.e., Trial Type: reward, non-reward) to form the PPI interaction terms. The gPPI model included physiological and psychological regressors, as well as their interaction terms to describe the unique effect of these interactions above and beyond the main effect of seed time courses and reward conditions. Our contrasts of interest evaluated the reward condition alone. No additional preprocessing procedures were completed beyond what has been described above. Supplemental analyses examined functional connectivity with anatomically defined right and left NAcc using the same procedures described for the functional connectivity analyses (see Additional file 1: Supplementary Materials).

#### Symptom analyses

Symptom analyses examined interactions between ASD symptom severity, measured by the Social Responsiveness Scale (SRS) [45], and functional activation and connectivity in the OT relative to PLC condition, conducted by including demeaned SRS values as a covariate within frontostriatal general linear models within the ASD group. Supplementary analyses examined interactions between ASD symptoms and structural activation, as well as functional activation of structurally defined clusters (see Additional file 1: Supplementary Materials).

### Salivary analyses

Saliva samples were collected using pediatric oral swabs (Salimetrics) prior to each nasal drug administration (i.e., OT and PLC) and immediately following the fMRI scan (time between samples in minutes *M* = 85; SD = 9). During each sample, participants were asked to place the swab under their tongue for approximately 1 min or until it was saturated with saliva. Samples were stored on ice for up to 2 h to liquid extraction and were permanently stored at − 70 °C (see Additional file 1: Supplementary Materials for a more detailed description of the salivary analyses).

## Results

### Face image ratings

**Fig. 1:**
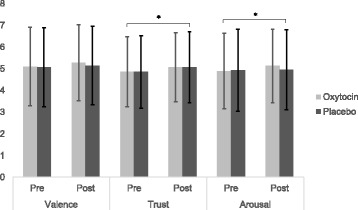
Subjective ratings of faces. Average ratings of valence, arousal, and trust of faces. Valence = 0 (extremely unpleasant) to 8 (extremely pleasant); arousal = 0 (not at all aroused) to 8 (extremely aroused); trust = 0 (not at all trustworthy) to 8 (extremely trustworthy). *p < .05

Participants rated the faces seen in the social reward condition on the dimensions of valence, arousal, and trust prior to and immediately following each scan. Results from a 2 (Treatment Group: OT, PLC) × 2 (Time point: pre- or post-scan) ANOVA revealed a main effect of time point for the dimension of trust, such that participants were more likely to rate the faces as more trustworthy at the post-scan rating (*M* = 5.07; SD = 1.59) compared to the pre-scan rating (*M* = 4.86; SD = 1.63), regardless of treatment condition, *F*(1,54) = 8.37, *p* = .006 (see Fig. 1). Additionally, a main effect of time point for the dimension of arousal was observed, reflecting that participants perceived the faces at the post-scan rating (*M* = 5.04; SD = 1.75) to be more arousing than those at the pre-scan rating (*M* = 4.91; SD = 1.80) across treatment groups, *F*(1,54) = 4.42, *p* = .040. No other main effects or interactions between treatment group and time point for the perceived valence, arousal, or trust of the faces were significant, all *p* values > .05.

### Task reaction times

**Fig. 2:**
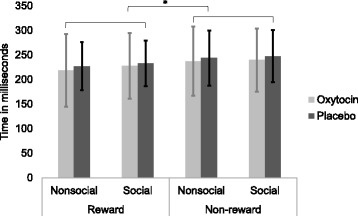
fMRI task reaction times. Mean reaction times of reward and non-reward trials during the social and nonsocial reward tasks. *p < .05

Reaction times (RTs) to task bull’s-eyes are depicted in Fig. 2 and were evaluated via a 2 (Treatment Group: OT, PLC) × 2 (Reward Condition: nonsocial, social) × 2 (Trial Type: reward, non-reward) mixed ANOVA. There was a main effect for trial type, *F*(1,54) = 18.67, *p* < .0001, such that individuals responded more quickly to trials during which they could receive a reward (*M* = 226.49; SD = 59.73) compared to trials in which they could not receive a reward (*M* = 242.03; SD = 60.91). No other main effects or interactions between treatment group, reward condition, and trial type were significant, all *p* values > .05.

### Functional activation analyses

#### Nonsocial reward

**Fig. 3:**
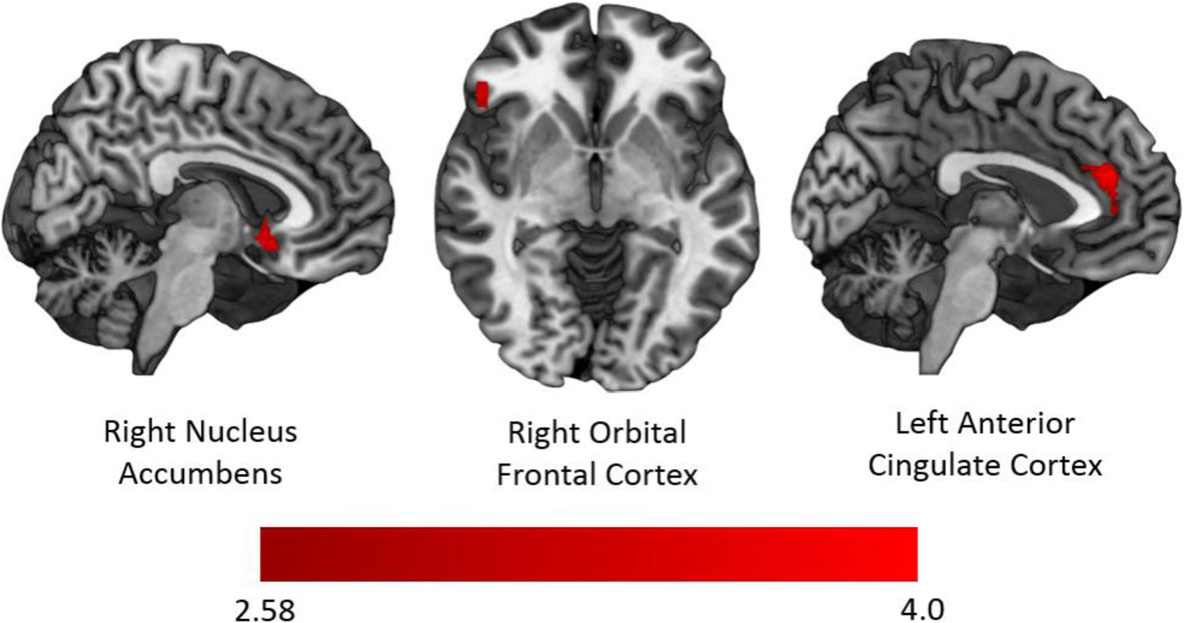
Differential functional activation after OT relative to PLC administration during nonsocial reward anticipation. Brain areas with greater activation during nonsocial reward anticipation after intranasal OT administration relative to PLC administration include the right nucleus accumbens (left), the right orbital frontal cortex (center), and the left anterior cingulate cortex (right)

**Fig. 4:**
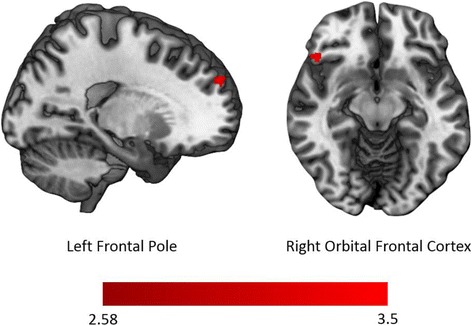
Differences in functional activation after OT relative to PLC administration during nonsocial reward outcomes. Brain areas with greater activation during nonsocial reward outcome after intranasal OT administration relative to PLC administration include the left frontal pole (left) and the right orbital frontal cortex (right)

During nonsocial reward anticipation, there were no regions with relatively decreased activation in the OT relative to the PLC condition. However, there were several clusters with greater activation during nonsocial reward anticipation in the OT condition relative to PLC, including the right NAcc, right frontal pole (FP), left ACC, left superior frontal cortex, and bilateral orbital frontal cortex (OFC) (see Fig. 3 and Table 2).^1^ Significant increases in activation were observed during nonsocial reward outcomes after OT relative to PLC administration in the right OFC and left FP (see Fig. 4).

**Table 2:** Effects of oxytocin on functional activation

Phase	Reward condition	Region	Hem	*k*	BA	*x*	*y*	*z*	Z max
OT > PLC									
Anticipation	Nonsocial	Frontal pole	R	316	10	41	95	38	3.99
		Anterior cingulate cortex	L	182	32	46	83	46	3.97
		Superior frontal cortex	L	83	–	49	74	65	3.31
		Orbital frontal cortex	R	76	–	23	79	30	3.26
			L	52	–	58	72	27	3.16
		Nucleus accumbens	R	56	–	43	72	30	3.42
	Nonsocial > social	Anterior cingulate cortex	L	441	–	46	84	46	3.91
		Frontal pole	L	69	–	54	91	29	3.12
			R	40	–	38	87	56	3.17
		Insular cortex	R	65	47	25	72	31	3.24
		Caudate nucleus	R	64	–	42	67	36	3.11
		Orbital frontal cortex	R	47	–	29	76	29	3.34
		Nucleus accumbens	R	21	–	42	71	30	3.09
Outcome	Nonsocial	Frontal pole	L	42		55	91	52	3.28
		Orbital frontal cortex	R	39	–	21	75	30	3.35
OT < PLC									
Outcome	Social	Frontal pole	R	50	–	24	91	37	3.29

*Hem* hemisphere, *k* cluster size in voxels, *BA* Brodmann area; *Z max* maximum *z*-value

Supplementary analyses for OT and PLC conditions separately are presented in Additional file 1: Supplementary Materials and visualized within Additional file 2: Figure S1 and Additional file 3: Figure S2. These simple effects analyses revealed that both groups showed activation in mesocorticolimbic reward processing regions in response to the social and nonsocial incentive delay tasks.

#### Social reward

During social reward outcomes, there was significantly decreased activation in the right frontal pole in the OT condition relative to the PLC condition. There were no other clusters with significant changes in activation during social anticipation or social outcomes in the OT condition relative to the PLC condition (see Additional file 1: Supplementary Materials and Additional file 4: Figure S3 for structural activation results for social and nonsocial reward anticipatory and outcomes).

#### Treatment Group × Reward Condition Interaction

We next evaluated the impact of OT, relative to PLC, on nonsocial versus social reward processing by evaluating a Treatment Group × Reward Condition interaction general linear model. OT increased activation in the right caudate nucleus, left ACC, bilateral FP, right insular cortex, and right OFC in response to nonsocial compared to social reward outcomes (see Table 2). Planned analyses within the striatal small volume revealed greater activation during nonsocial relative to social reward anticipation after intranasal OT relative to PLC in the right NAcc. There were no regions with greater activation during social relative to nonsocial reward anticipation or outcomes after intranasal OT relative to PLC.

#### Correlations between functional activation and ASD symptoms

**Fig. 5:**
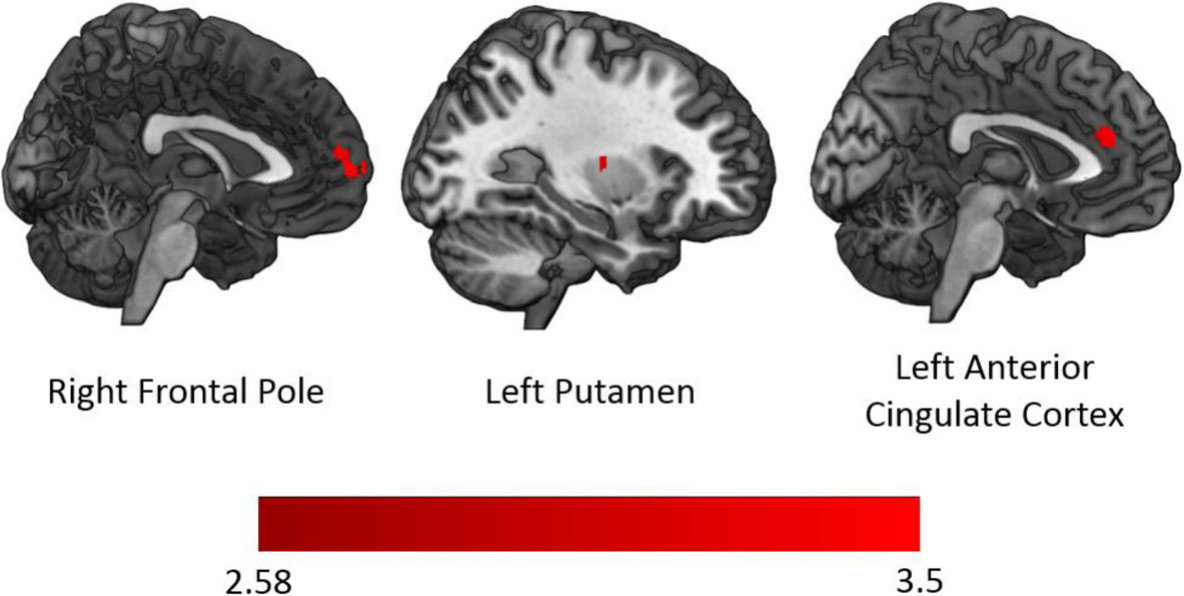
Correlations between SRS and differences in functional activation after OT vs. PLC during nonsocial reward anticipation. The right frontal pole, left putamen, and left anterior cingulate cortex showed increased activation in individuals with greater ASD symptoms during nonsocial reward anticipation following OT relative to PLC administration

Increased ASD symptom severity, as measured by SRS total scores, was associated with greater activation in the right FP and the right ACC during nonsocial reward anticipation and greater activation in the right precentral gyrus and left caudate nucleus during nonsocial reward outcome following the administration of OT relative to PLC (see Fig. 5 and Table 3). This finding within the left caudate nucleus was corroborated by structural activation analyses (see Additional file 1: Supplementary Materials). There were no relations between symptom severity and brain activation in the anticipation or outcome phases of the social reward condition.

**Table 3:** Correlations between ASD symptoms and functional activation to oxytocin relative to placebo

Phase	Reward condition	Region	Hem	*k*	BA	*x*	*y*	*z*	Z max
Anticipation	Nonsocial	Frontal pole	R	95	10	41	95	38	3.53
		Anterior cingulate cortex	L	82	32	46	83	46	3.41
Outcome	Nonsocial	Precentral gyrus	R	48	–	18	59	51	3.29
		Caudate nucleus	L	51	–	58	63	47	3.27

*Hem* hemisphere, *k* cluster size in voxels, *BA* Brodmann area, *Z max* maximum *z*-value

### Functional connectivity analyses

Given the prominent roles of the NAcc and ACC in reward processing [46, 47], functional connectivity analyses were seeded by the right NAcc and left ACC functional clusters that showed increased activation to OT relative to PLC during nonsocial reward anticipation in the functional activation analyses. Because there were no clusters that differentiated conditions in the social reward condition, functional connectivity results are only reported for connectivity in the nonsocial reward condition (functional connectivity of structurally defined clusters is presented in Additional file 1: Supplementary Materials).

#### Right nucleus accumbens seed

**Fig. 6:**
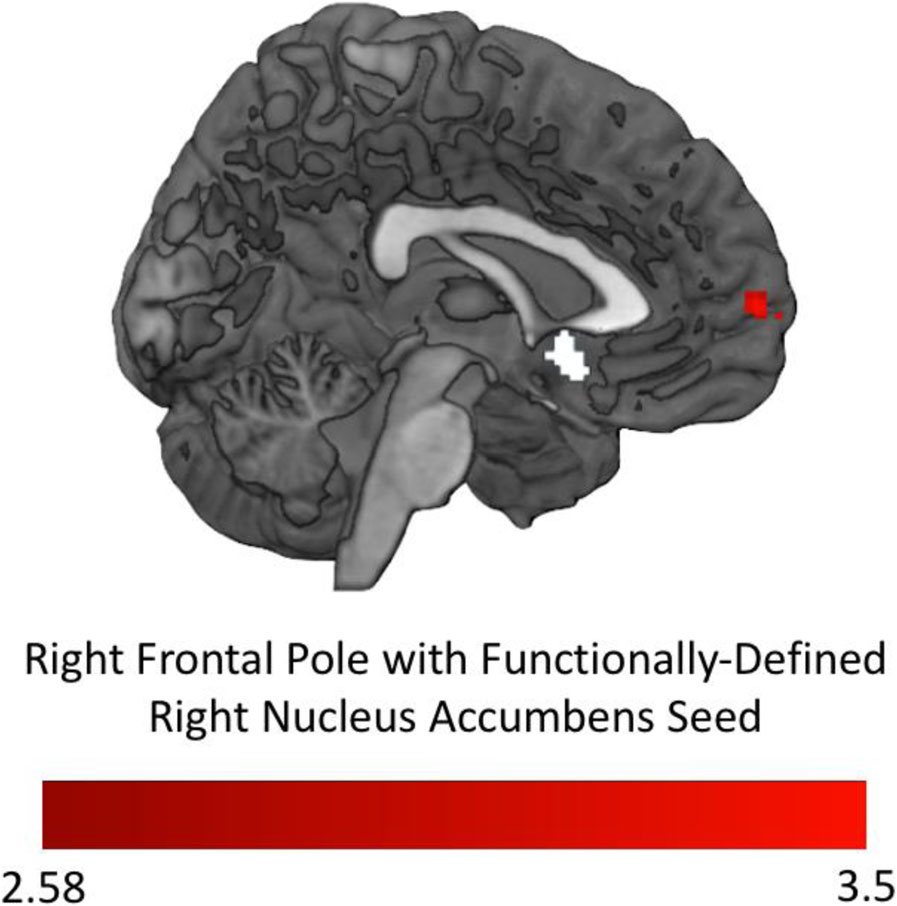
Functional connectivity during nonsocial reward anticipation with the functionally defined right nucleus accumbens seed. The right frontal pole (red) shows greater functional connectivity with the right NAcc (white) during nonsocial reward anticipation after intranasal OT administration relative to PLC administration

During nonsocial reward anticipation, OT relative to PLC administration resulted in increased functional connectivity between the right NAcc seed and the right frontal pole (see Fig. 6), whereas OT-induced decreases in functional connectivity were observed between the right NAcc seed and the left precentral gyrus and the right superior frontal gyrus (see Table 4). These findings were further corroborated by functional connectivity analyses of structurally defined clusters using a structural right NAcc seed (see Additional file 1: Supplementary Materials and Additional file 5: Figure S4). During nonsocial reward outcomes, increased functional connectivity was observed between the right NAcc and the right OFC and left FP in response to OT relative to PLC. Finally, decreased functional connectivity was exhibited between the right NAcc and right postcentral gyrus during nonsocial reward outcomes following OT administration relative to PLC.

**Table 4:** Functional connectivity with the right NAcc seed

Phase	Reward condition	Region	Hem	*k*	BA	*x*	*y*	*z*	Z max
OT > PLC									
Anticipation	Nonsocial	Frontal pole	R	45	–	39	95	39	3.39
Outcome		Orbital frontal cortex	R	82	–	22	75	31	3.96
		Frontal pole	L	41	9	54	92	52	3.19
OT < PLC									
Anticipation	Nonsocial	Precentral gyrus	L	266	–	54	62	63	3.64
		Superior frontal gyrus	R	53	–	37	63	69	3.6
Outcome		Postcentral gyrus	R	42	–	19	58	51	3.4

*Hem* hemisphere, *k* cluster size in voxels, *BA* Brodmann area, *Z max* maximum *z*-value

#### Anterior cingulate cortex seed

During the anticipation of nonsocial rewards, there was decreased functional connectivity between the left ACC and the left precentral gyrus, the right frontal pole, and the right superior frontal gyrus after OT relative to PLC. Attenuated functional connectivity with the left ACC was also observed with bilateral postcentral gyrus, the left inferior frontal gyrus, the left precentral gyrus, and the left medial frontal gyrus during nonsocial reward outcomes following OT relative to PLC (Table 5). No increases in connectivity were exhibited with the left ACC for nonsocial reward anticipation or outcomes, all *p* values > .05.

**Table 5:** Functional connectivity with the left ACC seed

Phase	Reward condition	Region	Hem	*k*	BA	*x*	*y*	*z*	Z max
OT < PLC									
Anticipation	Nonsocial	Precentral gyrus	L	206	–	58	58	63	3.86
		Frontal pole	R	197	–	30	83	48	3.34
		Superior frontal gyrus	R	39	–	37	63	69	3.66
Outcome		Postcentral gyrus	R	179	–	21	57	51	3.72
			L	90	3	75	57	50	3.13
		Inferior frontal gyrus	L	55	–	70	78	42	3.64
		Precentral gyrus	L	49	–	73	64	54	3.13
		Medial frontal gyrus	L	42	6	57	66	59	3.09

*Hem* hemisphere, *k* cluster size in voxels, *BA* Brodmann area, *Z max* maximum *z*-value

#### Correlations between functional connectivity and ASD symptoms

For the right NAcc and left ACC seeds, greater ASD symptom severity, measured by SRS total scores, was associated with increased connectivity with the right postcentral gyrus during nonsocial reward outcomes following OT relative to PLC (see Table 6). During nonsocial reward anticipation, there were no significant correlations between SRS scores and connectivity with the right NAcc or left ACC following OT relative to PLC.

**Table 6:** Correlations between ASD symptoms and functional connectivity for oxytocin relative to placebo

Phase	Reward condition	Region	Hem	*k*	BA	*x*	*y*	*z*	Z max
Right NAcc seed									
Outcome	Nonsocial	Postcentral gyrus	R	74	–	18	58	51	3.37
Left ACC seed									
Outcome	Nonsocial	Postcentral gyrus	R	131	–	18	58	51	3.5

*Hem* hemisphere, *k* cluster size in voxels, *BA* Brodmann area, *Z max* maximum *z*-value

### Salivary OT

**Fig. 7:**
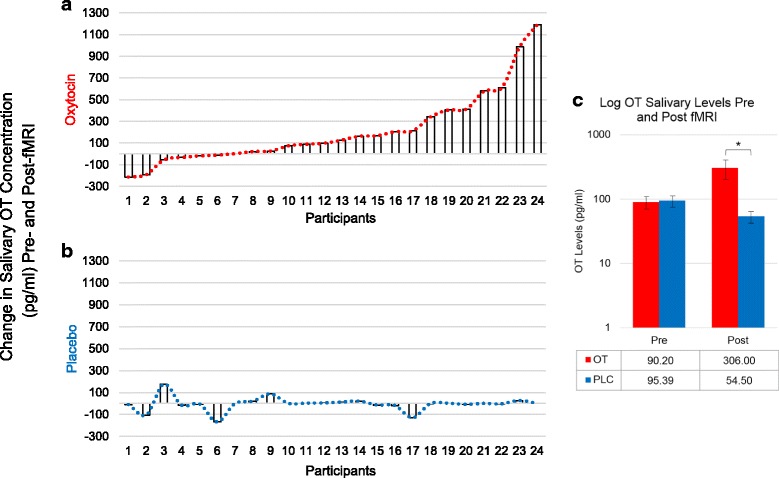
Salivary OT concentrations. Change in log-transformed salivary OT levels (pg/ml) for 24 participants (minutes between samples *M* = 85; SD = 9). Four participants were unable to provide adequate saliva samples and were not included in the salivary analyses. **a** Change in salivary OT following nasal OT administration. **b** Change in salivary OT following nasal-PLC administration. Because participant 10 was a significant outlier (change in OT concentration after PLC = − 723.59), their data are not included in the graph above. **c** *p < .05. Salivary samples collected after OT administration showed significantly greater OT concentrations compared to those following the PLC nasal spray, *t* = 3. 57; *p* = 0.0016

To examine changes in OT concentration levels, salivary samples were collected prior to OT administration and immediately following the fMRI scan. There were considerable individual differences in the magnitude of salivary OT change from baseline to post-scan following OT administration, and, thus, one outlier was removed from salivary analyses due to significantly elevated OT concentration levels (754.17 pg/ml) in the PLC condition. After the removal of this outlier, as expected, there was a significant increase in mean peripheral OT levels following OT administration relative to PLC, *t* = 3.57; *p* = 0.0016 (see Fig. 7).

**Fig. 8:**
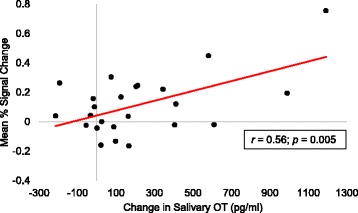
Correlations between OT-related neural activation and OT salivary concentration changes following OT administration. Correlation between mean percent signal change in the right NAcc functional activation cluster during the anticipatory phase of the nonsocial reward condition and change in peripheral OT levels following OT administration

Because of the primary role of the NAcc in reward processing [46], correlation analyses examined relations between changes in peripheral OT and neural activation within the right NAcc functional activation cluster identified in the nonsocial anticipation activation analysis. This revealed a significant positive correlation indicating that individuals with greater changes in peripheral OT concentrations following OT administration showed greater increased activation within the right NAcc functional activation cluster during nonsocial reward anticipation, *r* = 0.56; *p* = 0.005 (see Fig. 8). However, when a significant outlier (2 SD’s > the salivary group mean; 3 SD’s > the activation group mean) was removed, the relation was no longer significant, *r* = 0.26; *p* = 0.23.^2^

## Discussion

The purpose of this investigation was to examine the effects of acute intranasal OT administration on functional activation and connectivity within mesocorticolimbic brain regions during the anticipation and receipt of social and nonsocial rewards in ASD. OT administration, relative to PLC administration, was associated with increased activity in the right NAcc, the right FP, the left ACC, the left superior frontal cortex, and bilateral OFC during anticipation of nonsocial rewards. These findings combined with prior ASD research demonstrating increased activation in the NAcc following OT administration during a social judgment task [22] suggest that whether OT impacts social or nonsocial processing is contingent on task context. In addition, the correlation between salivary OT concentrations and changes in right NAcc activation indicates that this region may be particularly sensitive to the acute effects of OT (though this correlation was not significant following removal of an outlier). This is consistent with preclinical findings, which indicate that the NAcc is among several neural regions with the highest OT receptor density [48].

Although we found increased left ACC activation after OT administration during nonsocial reward anticipation, Watanabe and colleagues [49] reported increased ACC activation after OT administration during a social judgment task, reflecting the task-dependent nature of the effects of OT on neural responses to social or nonsocial processing. Our finding of increased activation of OFC, a region with an established role in reward processes documented in preclinical and clinical studies [50, 51], during the anticipation and receipt of nonsocial rewards after OT administration is consistent with prior findings that ASD is characterized by attenuated OFC activation during nonsocial reward anticipation [26] and suggests a remediation of this pattern in ASD after OT.

In contrast to previous studies examining the neural impact of OT in response to social stimuli in individuals with ASD [22, 23], we did not find evidence of increased activity in mesocorticolimbic regions during social reward processing following OT administration. Further, interaction analyses showed increased activity in the right nucleus accumbens and right caudate nucleus during nonsocial reward anticipation relative to social reward anticipation. The lack of effects of OT in the social reward conditions are surprising and stand in contrast to preclinical findings that OT enhances neural responses to a range of social stimuli, including conditioned social preference [52–54] and reproductive behaviors [55, 56] as well of the prosocial effects of OT in ASD [57]. These unexpected findings highlight that OT may serve to increase neural activations in response to nonsocial rewards. These effects are consistent with preclinical findings that the impact of OT is apparent in the context of a certain nonsocial rewards, including food cues [58, 59] and place preferences [60, 61], and it may be the case that the clinical benefits of OT on social functioning in ASD (e.g., enhanced emotion recognition and increased eye gaze) reflect the influence of OT on mesocorticolimbic reward processing systems that mediate nonsocial incentive salience processing, reward valuation, and reward-based learning [62] rather than responses specifically to social rewards. Alternatively, it may be the case that the static social rewards used in this study impeded our capacity to detect OT-related neural changes given that dynamic stimuli have been shown to be more potent elicitors of social impairments in ASD than static stimuli [63]. Future studies that evaluate the impact of OT on neural responses to dynamic social rewards will be needed to evaluate this possibility.

We observed significant correlations between ASD symptom severity and increased activity within the right frontal pole and the left ACC during nonsocial reward anticipation in response to OT relative to PLC. Additionally, during nonsocial reward outcomes, increases in the left caudate nucleus and right precentral gyrus activity after OT relative to PLC were significantly correlated with symptom severity. The postcentral gyrus also showed greater connectivity with both the right NAcc and left ACC functional seeds as ASD symptom severity increased. These regions may be most responsive to neural effects of OT administration in individuals with more severe ASD presentations. Alternatively, these associations suggest that the impact of OT on responses to nonsocial rewards may be conditional on ASD symptom severity. These associations may also reflect mechanisms described by Parker and colleagues [15] which revealed that individuals with ASD with lower endogenous levels of OT benefited the most from OT. Thus, it may be the case that individuals with greater ASD symptoms demonstrated greater regional activation changes during reward anticipation in response to OT. It is noteworthy that symptom correlations with neural responses to nonsocial reward anticipation were apparent in brain regions (FP and ACC) implicated in higher-order executive processing [64] and known to show functional impairments in ASD in the context of cognitive control tasks [65, 66]. Conversely, regions showing symptom correlations with neural responses to social reward anticipation involved regions implicated in other functioning, including imitation (precentral gyrus [67]) and learning (the caudate nucleus [68]), though the replicability of these patterns is not yet known.

OT administration was associated broadly with decreased connectivity with functional seeds. Decreased connectivity was observed between the right NAcc and the left precentral gyrus and the right superior frontal gyrus during the anticipation of nonsocial rewards as well as with the postcentral gyrus during nonsocial reward outcomes following OT administration relative to PLC. Further, OT-induced attenuation in functional connectivity was observed between the left ACC functional seed and the left precentral gyrus, the right frontal pole, and the right superior frontal gyrus during nonsocial reward anticipation. During nonsocial reward outcomes, decreased functional connectivity was observed between the left ACC and bilateral postcentral gyrus, left inferior frontal gyrus, left precentral gyrus, and left medial frontal gyrus following OT relative to PLC. Resting state functional connectivity findings suggest that ASD is largely characterized by increased frontostriatal connectivity relative to typically developing controls [69–72], and the results of the present study suggest that OT may normalize these increased frontostriatal functional connections.

There were additional findings of increased functional connectivity after OT administration, including increased connectivity between the right NAcc and the right FP during nonsocial reward anticipation. OT-induced increased connectivity between the right NAcc and right FP was also reported by Gordon et al. [23] using a biological motion task. This finding across two different task contexts highlights a neural pathway by which OT may exert a therapeutic effect by potentiating neural connectivity. The FP plays a critical role in the cognitive processing of future events [73], a process that may be particularly relevant to reward contexts. Additionally, the right NAcc demonstrated relatively greater connectivity with the right FP and right OFC during nonsocial reward outcomes following OT administration relative to PLC, though the directionality of this effect was unexpected given that increased functional connectivity between the striatum and the OFC has been reported in ASD during resting-state functional connectivity [69]. It is also noteworthy that the effects of OT on the NAcc and ACC exhibited right-lateralized effects given evidence of right lateralization of functional neural responses to social and nonsocial stimuli in ASD [74, 75], though it should be noted that incentive delay tasks do not reliably evoke greater activation in one hemisphere or the other but rather tend to evoke bilateral reward-related frontostriatal activations [76].

Ratings of faces in the social task revealed a significant increase in ratings of trustworthiness and arousal for faces following the scan. These main effects were not moderated by treatment group (i.e., OT, PLC), indicating that individuals rated faces they had seen previously as more trustworthy across both treatment groups. Previous studies have reported that individuals with ASD reliably understand the concept of trustworthiness and distinguish trustworthy versus non-trustworthy faces [77, 78]. Our results suggest that familiarity with faces may increase ratings of trustworthiness and arousal for individuals with ASD. No effects were observed for ratings of valence.

Task reaction times showed increased speed of responses to reward relative to non-reward trials, with no significant interactions of treatment group (OT, PLC) or Reward Type (nonsocial, social). These findings are consistent with reports of decreased reaction times for reward compared to non-reward trial in ASD [79]. Delmonte and colleagues [79] reported no relation between reward condition (e.g., nonsocial vs. social) and reaction times. However, this stands in contrast with other ASD reward studies that have reported faster reaction times in response to nonsocial rewards compared to social rewards [26, 80]. This discrepancy may reflect different ages of participants across studies: the current study and others showing no differences in reaction times based on reward condition were conducted in child and adolescent populations, whereas those showing faster responses for nonsocial versus social rewards were completed using adult participants. This may suggest that during development, nonsocial rewards may begin to have increased salience relative to social rewards in individuals with ASD. This might be related to increased awareness of the relationship between money and acquiring objects of interest and/or to increased demands in financial responsibility for adults living independently. This developmental interaction should be noted in future studies examining differential responses to nonsocial versus social rewards in ASD. It may also be useful to explore the salience of other nonsocial rewards in ASD.

In addition to the substantive findings reported here, these results have implications for future experimental therapeutic trials that seek to evaluate novel ASD therapeutics. The National Institute of Health has emphasized the use of translational research to speed the discovery of treatments through pipelines that evaluate the potential for novel compounds to engage brain targets relevant to disease etiology [81]. In addition to providing substantive results about the neural impact of acute intranasal OT administration on reward processing brain systems, the present study also suggests that optimal approaches to evaluate novel ASD treatments with putative effects on brain systems that support social reward processing may not be constrained to evaluating responses to only social stimuli. Rather, novel pro-social ASD therapeutics may exert their influence on relevant brain targets in a range of social and/or nonsocial contexts. In this regard, these results provide preliminary data to guide the development of optimal targets for use in future experimental therapeutics trials that evaluate novel ASD social communication treatments.

The present study had some limitations. Developmental stage plays a particularly important moderating role in the strength of functional connectivity patterns in individuals with ASD, with younger individuals showing increased connectivity compared to adolescents and adults with ASD [82]. Future studies with large sample sizes will be needed to examine the moderating effect of developmental stage on the effects of OT on brain activation and connectivity in ASD. Additionally, the effects of prolonged OT administration are likely to be distinct from the effects of a single dose, and future research should examine the effect of chronic OT administration on neural functioning in ASD. Additionally, the order of social and nonsocial runs was not randomized across participants in this study. Because the current study found no behavioral changes due to a single OT administration, interpretations regarding associations between behavioral and neural effects of OT must be cautious. Finally, because all participants in the present study met a minimum IQ cutoff of 70, findings from this study may be restricted to individuals with ASD with higher cognitive ability.

## Conclusions

Despite these limitations, these findings indicate a mechanistic role for the mesocorticolimbic system in the potentially therapeutic effect of oxytocin in individuals with ASD. These findings align with prior studies that highlight the important role of enhanced functioning of striatal regions as a potential mechanism of action of OT [22, 23] and extend this area of research into the domain of striatal functioning in response to reward-based tasks. When the present findings are considered along with these prior fMRI studies, it appears that the role of the mesocorticolimbic system in the effects of OT on neural functioning is not confined to social rewards but may extend to nonsocial responses more broadly, depending on task contexts.

## Additional files

Additional file 1: Supplementary Analyses & Results. (DOCX 31 kb)

Additional file 2: **Figure S1.** Functional activation during the anticipatory phase of the nonsocial and social tasks for OT and PLC. (DOCX 281 kb)

Additional file 3: **Figure S2.** Functional activation during the outcome phase of the nonsocial and social tasks for OT and PLC. (DOCX 269 kb)

Additional file 4: **Figure S3.** Structural activation in striatal regions during the anticipation and outcome of nonsocial and social rewards. Frontostriatal structural activation during nonsocial (left) and social (right) reward anticipation and outcome after intranasal OT relative to PLC administration. In the nonsocial reward condition, the right NAcc showed relatively increased activation during reward anticipation following OT relative to PLC administration. No significant differences in activation were observed during nonsocial outcomes following OT relative to PLC administration. In the social reward conditions, none of the regions queried showed differential activation during either the anticipation or outcome phases after intranasal OT relative to PLC administration. NAcc = nucleus accumbens. *p < .05. (DOCX 57 kb)

Additional file 5: **Figure S4.** Functional connectivity with structurally defined right NAcc during nonsocial reward anticipation. The right frontal pole (red) shows greater structural connectivity with the right NAcc (white) during nonsocial reward anticipation after intranasal OT administration relative to PLC administration. (DOCX 1641 kb)

## Abbreviations

ACC: Anterior cingulate cortex
ADOS: Autism Diagnostic Observation Schedule
ASD: Autism spectrum disorder
FEAT: FSL expert analysis tool
fMRI: Functional magnetic resonance imaging
FP: Frontal pole
GLM: General linear model
gPPI: Generalized psychophysiological interaction
NAcc: Nucleus accumbens
OFC: Orbital frontal cortex
OT: Oxytocin
PLC: Placebo
ROI: Region of interest
SRS: Social Responsiveness Scale
WASI: Wechsler Abbreviated Scales of Intelligence

## Footnotes

1 Similar analyses during the anticipation of nonsocial rewards were conducted after removing both female participants, as well as a participant with outlying salivary OT concentrations (see Figure 7a). Results from these analyses remained statistically significant within all regions reported in Table 2. Therefore, all activation results include both females and the participant with outlying salivary OT levels.

2 We thank an anonymous reviewer for highlighting this. This outlier was not the same outlier as the one mentioned in the previous salivary analyses examining treatment group (OT vs. PLC) differences in OT concentrations.
