# Supplementary material for: The effects of intranasal oxytocin on reward circuitry responses in children with autism spectrum disorder
Source: J Neurodev Disord. 2018 Mar 27;10:12. doi: 10.1186/s11689-018-9228-y (PMC5870086; doi:10.1186/s11689-018-9228-y)
Supplement: Supplementary file 1 — Supplementary Analyses & Results. (DOCX 31 kb) [file 11689_2018_9228_MOESM1_ESM.docx]

**Supplementary Materials for “The Effects of Intranasal Oxytocin on Reward Circuitry Responses in Children with Autism Spectrum Disorder”**

**Supplementary Materials I. Main Effect of OT and PLC Separately on nonsocial and social tasks**

**Anticipation**. Whole brain functional activation during anticipation revealed increased neural activations within midbrain and striatal regions, as well as bilateral ACC, bilateral paracingulate gyrus, bilateral precentral gyrus, bilateral occipital pole, bilateral lingual gyrus, and cerebellum. This pattern was broadly observed during the anticipation of both social and nonsocial rewards following OT and PLC administrations to varying degrees of intensity (see Supplementary Figure 1).

**Outcome.** Whole brain functional activation during anticipation revealed increased neural responses in bilateral occipital pole, bilateral ACC, bilateral orbital frontal cortex, bilateral insular cortex, bilateral frontal pole, and striatal regions during nonsocial and social reward outcomes following OT or PLC administration (see Supplementary Figure 2). These findings indicate that neural activations largely demonstrated phase-specific (i.e., Anticipation, Outcome) patterns across Treatment Group (i.e., OT, PLC) and Reward Condition (i.e., Nonsocial, Social).

**Supplementary Materials II. Structural Activation Analyses & Results**

For each ROI and participant, treatment-group- and run-specific mean parameter estimates reflecting activation were calculated and extracted using the Featquery tool within FSL separately for anticipation and outcome phases of the social and nonsocial tasks. A 2 (Treatment Group: OT, PLC) × 2 (Reward Condition: Nonsocial, Social) ANOVA was conducted for each of the six striatal regions of interest (right/left NAcc, right/left caudate nucleus, and right/left putamen). No significant Treatment Group × Reward Condition interactions were found. Because of the significant functional activation differences seen between treatment groups, paired t-tests were employed to examine structural activation differences within the six striatal regions of interest. These analyses were conducted separately for nonsocial and social anticipation and outcomes.

**Nonsocial Reward Condition.** During nonsocial reward anticipation, results revealed significant treatment group differences in structural activation within the right NAcc, *t*(27) = 2.56, *p* = 0.017, with increased activation associated with OT administration as compared to PLC (see the left side of Supplementary Figure 3). No other significant treatment group differences in structural activation were found within either the anticipatory or outcome phase analyses.

**Social Reward Condition.** No significant treatment group differences in structural activation were found during either social anticipation or outcomes, all *p’s >* .05 (see the right side of Supplementary Figure 3).

**Supplementary Materials III. Functional Connectivity of Structurally-Defined Clusters Analyses & Results**

**Right Nucleus Accumbens.**

***Nonsocial Task.*** During nonsocial reward anticipation, the OT group exhibited increased connectivity, relative to PLC, between the right NAcc seed and right frontal pole (see Supplementary Figure 4). Additionally, during nonsocial reward anticipation, decreased connectivity was seen between the right NAcc seed and the left superior frontal gyrus for OT compared to PLC. Treatment group differences in task-dependent structural connectivity are illustrated in Supplementary Table 1.

***Social Task.*** During social reward anticipation, the OT group showed significant decreased connectivity between the right NAcc seed and the left caudate nucleus. There were no significant increased effects of OT with connectivity during either social or nonsocial outcomes.

*Functional Connectivity of Structurally-Defined Clusters*

| **Phase** | **Reward Condition** | **Region** | **Hem** | **k** | **BA** | **x** | **y** | **z** | **Z max** |
| --- | --- | --- | --- | --- | --- | --- | --- | --- | --- |
| **Right Nucleus Accumbens Seed** | | | | | | | | | |
| OT > PLC | | | | | | | | | |
| Anticipation | Nonsocial | Frontal Pole | R | 101 | -- | 40 | 95 | 38 | 3.73 |
| OT < PLC | | | | | | | | | |
| Anticipation | Nonsocial | Superior Frontal Gyrus | L | 49 | -- | 54 | 62 | 63 | 3.46 |
|  | Social | Caudate Nucleus | L | 47 | -- | 52 | 61 | 48 | 3.5 |
| **Left Nucleus Accumbens Seed** | | | | | | | | | |
| OT > PLC | | | | | | | | | |
| Anticipation | Nonsocial | Frontal Pole | R | 76 | -- | 40 | 95 | 38 | 3.61 |
|  |  | Anterior Cingulate Cortex | L | 58 | 32 | 46 | 83 | 46 | 3.43 |
| Outcome | Nonsocial | Orbital Frontal Cortex | R | 120 | -- | 22 | 75 | 31 | 3.42 |
| OT < PLC | | | | | | | | | |
| Anticipation | Nonsocial | Precentral Gyrus | L | 117 | -- | 57 | 58 | 63 | 3.53 |
|  | Social | Frontal Pole | R | 54 | -- | 24 | 88 | 37 | 3.23 |
|  |  | Caudate Nucleus | L | 44 | -- | 52 | 61 | 48 | 3.47 |
| Outcome | Nonsocial | Precentral Gyrus | L | 40 | -- | 71 | 67 | 53 | 3.12 |

Supplementary Table 1. Frontostriatal structural connectivity clusters showing treatment group differences (minimum cluster size = 39 voxels) with the right and left NAcc as structural seeds. Hem=Hemisphere; k=cluster size in voxels; BA=Brodmann Area; Z max=maximum z-value.

**Left Nucleus Accumbens.**

***Nonsocial Reward Condition.*** The OT group exhibited increased connectivity, relative to PLC, between the left NAcc seed and right FP and left ACC during nonsocial reward anticipation (see Supplementary Table 1). Increased connectivity was also observed in the right OFC during nonsocial reward outcomes following OT relative to PLC. Finally, our findings revealed OT-induced decreases in connectivity in the left precentral gyrus during nonsocial reward anticipation and outcomes when compared to PLC.

***Social Reward Condition.*** Decreased connectivity was also observed in the right FP and left caudate nucleus during social reward anticipation following OT compared to PLC. There were no effects of OT relative to PLC on left NAcc connectivity in social reward outcomes in frontostriatal regions.

**Supplementary Materials IV. Correlations Between Structural Activation and ASD symptoms**

Increased ASD symptom severity, as measured by SRS total scores, was associated with greater activation in the left caudate nucleus during nonsocial reward outcome following the administration of OT, *r*(26) = .43, *p* = .023. Additionally, findings from correlational analyses between SRS total scores and activation in response to nonsocial reward outcome within the right caudate nucleus, *r*(26) = .37, *p* = .052, and left putamen, *r*(26) = .35, *p* = .066, trended toward significance. There were no significant relations between symptom severity and brain activation during the anticipation of nonsocial rewards or the anticipation or outcome of social rewards.

**Supplementary Materials V. Correlations Between Functional Connectivity of Structurally-Defined Clusters and ASD symptoms**

Additionally, increased ASD symptoms were associated with greater functional connectivity between the structurally-defined right NAcc seed and the right FP during the anticipation of nonsocial rewards. For both the structurally-defined right and left NAcc seeds, increased connectivity with the right postcentral gyrus was associated with more severe symptomatology during the outcome of nonsocial rewards. Finally, increased connectivity between the right NAcc seed and the right paracingulate gyrus was associated with greater ASD severity in response to social reward outcomes. There were no significant relations between symptom severity and functional connectivity of structurally-defined left NAcc during nonsocial anticipation or social anticipation or outcomes. Similarly, there were no significant associations between functional connectivity of structurally-defined right NAcc and ASD behavioral measures during the anticipation of social rewards.

**Supplementary Materials VI. Salivary Analysis Methods**

All samples were extracted prior to oxytocin analysis using strata-X 33µm polymeric reversed phase SPE sorbent in a 96-well plate containing 60 mg sorbent per well, Phenomenex, Torrance CA. Plasma and saliva were acified with 1.5% trifluoroacetic acid (TFA) and centrifuged at 6,000 x g for 20 minutes at 4°C. The supernatant was loaded onto an activated strata-X plate. Wells were washed with 1.5 ml of 0.1% TFA, and then the oxytocin peptide eluted with 1ml of 80% acetonitrile. The eluant was evaporated to dryness under a N2 stream and reconstituted in 250ul of assay buffer. Extraction efficiency was determined by spiking a sample with a known amount of hormone and extracting with the other samples (typically > 90%).

Oxytocin levels in extracted saliva were measured using an assay kit and protocol from Enzo Life Sciences, Ann Arbor, MI. The endogenous OT hormone competes with oxytocin linked to alkaline phosphatase for the oxytocin antibody binding sites. After the overnight incubation at 4°C, the excess reagents are washed away and the bound oxytocin phosphatase was incubated with substrate and after 1 hour this colormetric enzyme reaction was stopped. The hormone content (pg/ml) is determined by plotting the OD of each sample against a standard curve. The sensitivity of the assay is 2.4 pg/ml with a standard range of 5 to 320 pg/ml. The intra- and inter- assay variations are 4.8% and 8% respectively. Enzo Life Sciences reports cross-reactivity for similar neuropeptides found in mammalian sera at less than 0.001.
