# Supplementary figures and images for: The effects of intranasal oxytocin on reward circuitry responses in children with autism spectrum disorder
Source: J Neurodev Disord. 2018 Mar 27;10:12. doi: 10.1186/s11689-018-9228-y (PMC5870086; doi:10.1186/s11689-018-9228-y)

­­­
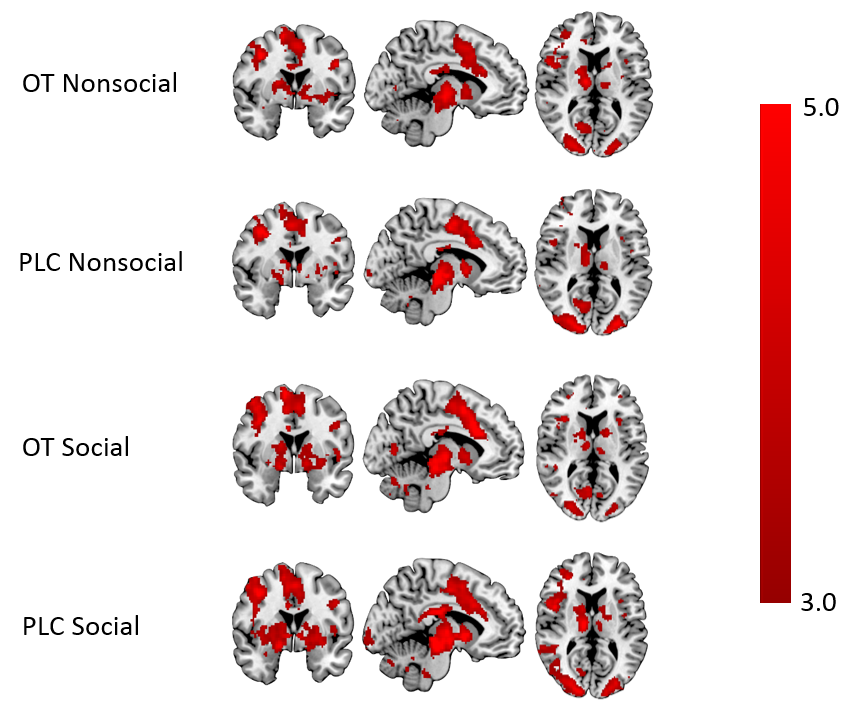

Supplement: Supplementary file 2 — Figure S1. Functional activation during the anticipatory phase of the nonsocial and social tasks for OT and PLC. (DOCX 281 kb) [file 11689_2018_9228_MOESM2_ESM.docx]

­­­
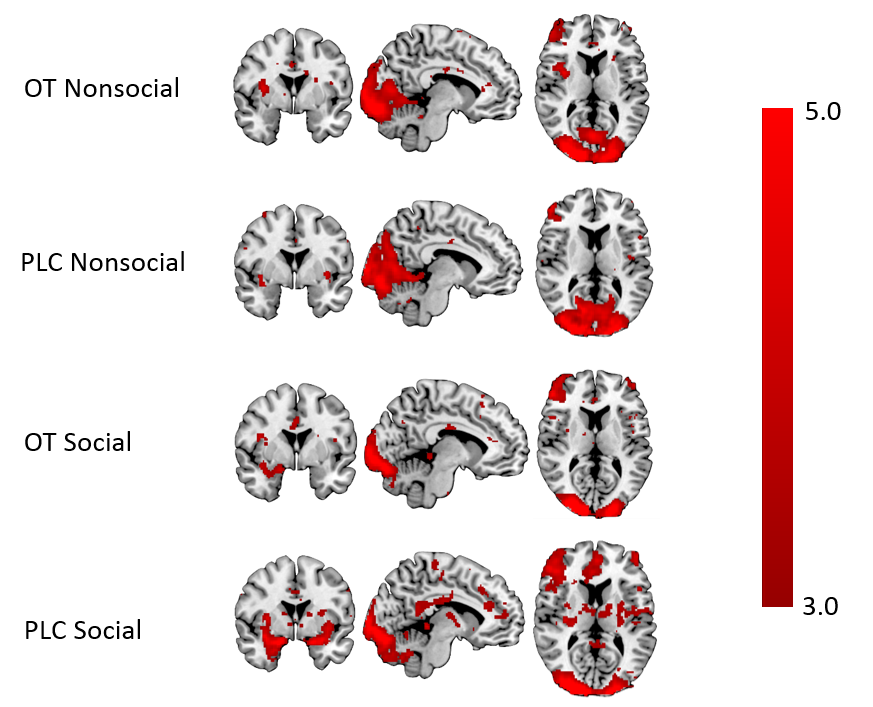

Supplement: Supplementary file 3 — Figure S2. Functional activation during the outcome phase of the nonsocial and social tasks for OT and PLC. (DOCX 269 kb) [file 11689_2018_9228_MOESM3_ESM.docx]

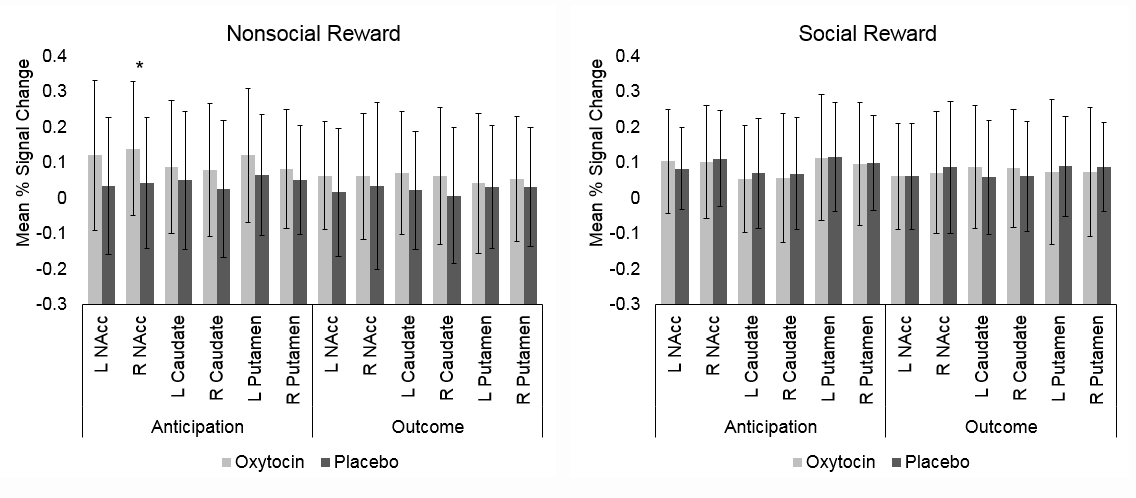

Supplement: Supplementary file 4 — Figure S3. Structural activation in striatal regions during the anticipation and outcome of nonsocial and social rewards. Frontostriatal structural activation during nonsocial (left) and social (right) reward anticipation and outcome after intranasal OT relative to PLC administration. In the nonsocial reward condition, the right NAcc showed relatively increased activation during reward anticipation following OT relative to PLC administration. No significant differences in activation were observed during nonsocial outcomes following OT relative to PLC administration. In the social reward conditions, none of the regions queried showed differential activation during either the anticipation or outcome phases after intranasal OT relative to PLC administration. NAcc = nucleus accumbens. *p < .05. (DOCX 57 kb) [file 11689_2018_9228_MOESM4_ESM.docx]

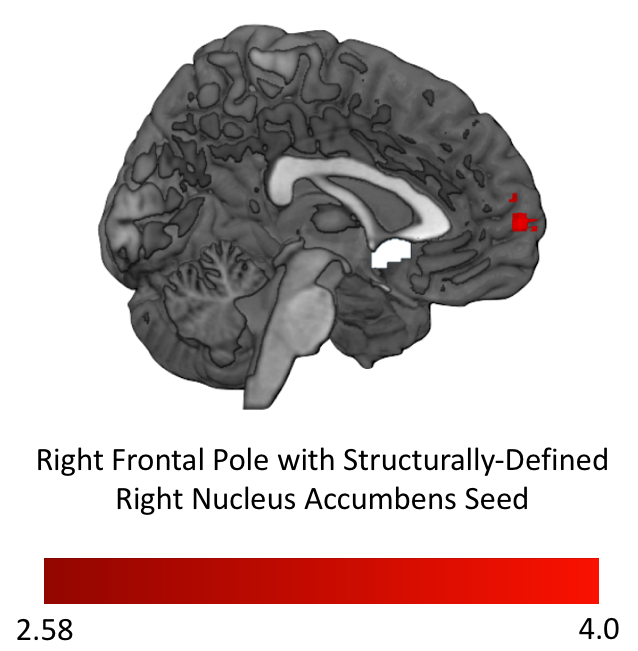

Supplement: Supplementary file 5 — Figure S4. Functional connectivity with structurally defined right NAcc during nonsocial reward anticipation. The right frontal pole (red) shows greater structural connectivity with the right NAcc (white) during nonsocial reward anticipation after intranasal OT administration relative to PLC administration. (DOCX 1641 kb) [file 11689_2018_9228_MOESM5_ESM.docx]
